# Computer-assisted, template-guided immediate implant placement and loading in the mandible: a case report

**DOI:** 10.1186/s12903-019-0746-0

**Published:** 2019-04-11

**Authors:** Thomas Spielau, Uli Hauschild, Joannis Katsoulis

**Affiliations:** 1 Implantology, Oral Surgery; Private dental office, Johannesstrasse 7-9, 474623 Kevelaer, Germany; 2 Department of Postgraduate Education, Faculty of Oral and Dental Medicine at J.W, Goethe-University Frankfurt am Main, Frankfurt am Main, Germany; 3 Department of Reconstructive Dentistry and Gerodontology, School of Dental Medicine, University of Bern, Bern, Switzerland

**Keywords:** Virtual implant planning, Guided surgery, Immediate placement, Immediate loading, CAD/CAM

## Abstract

**Background:**

Computer-assisted implant planning has become an important diagnostic and therapeutic tool in modern dentistry. This case report emphasizes the possibilities in modern implantology combining virtual implant planning, guided surgery with tooth and implant supported templates, immediate implant placement and loading.

**Case presentation:**

A straight forward approach was followed for the mandible presenting with hopeless lower incisors. Diagnosis, decision making and treatment approach were based on clinical findings and detailed virtual three-dimensional implant planning. Extractions of the hopeless mandibular incisors, immediate and guided implant placement of six standard implants, and immediate loading with a provisional fixed dental prosthesis (FDP) were performed fulfilling patient’s functional and esthetic demands. The final computer assisted design / computer assisted manufacturing (CAD/CAM) FDP with a titanium framework and composite veneering was delivered after 6 months. At the 1-year recall the FDP was free of technical complications. Stable bony conditions and a healthy peri-implant mucosa could be observed.

**Conclusions:**

Computer assisted implantology including three-dimensional virtual implant planning, guided surgery, and CAD/CAM fabrication of provisional and final reconstructions allowed for a concise treatment workflow with predictable esthetic and functional outcomes in this mandibular full-arch case. The combination of immediate implant placement and immediate loading was considerably more complex and required a high level of organization between implantologist, technician and patient. After the usage of a first tooth-supported surgical template with subsequent extraction of the supporting teeth, a second surgical template stabilized on the previously inserted implants helped to transfer the planned implant position in the extraction sites with a guided approach.

## Background

Computer assisted implantology (CAI) was introduced more than 25 years ago and aimed to facilitate implant planning and to avoid intraoperative complications such as mandibular nerve damage, sinus perforations, fenestrations, or dehiscence [1–4]. Based on a computerized tomography (CT) scan and a digitized tooth setup, the prosthetically ideal implant positions can be planned virtually with the help of a guided surgery software allowing for three dimensional visualization prior to implant surgery [2, 5, 6]. Furthermore, the possibility to transfer the virtually planned implant position to the real clinical situation is provided by a stereolithographically fabricated surgical template [3, 7]. While only few guided implant placement systems were available at the time, today, multiple CAI software are available on the market. Several in-vitro, cadaver and clinical studies have reported on the accuracy of guided implant placement [8–10]. Although the current state of software and hardware technology has improved, inaccuracies in implant placement may occur and depend on different factors such as the template support (bone, mucosa, teeth, implants), intrinsic factors of the surgical guide (tolerance in diameter between the drill and the guide sleeve, fabrication accuracy of the guide) [11, 12] and human related factors during the workflow of virtual planning and guided surgery [7, 13]. The guided surgery approach is still controversially discussed [14–16] even though the procedure may be performed in a safe and predictable way [17, 18]. However, a systematic and concise approach performing the single steps in the treatment sequence may allow for more accurate implant positioning as type of guide and fixation have an important influence [19, 20]. Additionally, the use of multiple templates with different supports, i.e. teeth and implant support combined in a sequenced order is believed to improve accuracy compared to a mucosa supported approach alone [21].

While some patients wish to be informed in detail about the specific treatment steps, most of them want to know whether they would have to leave the dental office without teeth at some point of the treatment. In this context, immediate implant placement after tooth extraction and immediate implant loading with a fixed provisional reconstruction may help the patient as time after extractions and osseointegration is consolidated. In guided surgery protocols, minimally invasive placement and immediate loading has been a possible treatment step from the beginning [3, 4]. Postoperative morbidity after flapless surgery is significantly reduced compared to the traditional open approach, especially in edentulous patients [17, 22, 23]. Later during the treatment, reconstructions fabricated with the help of computer assisted design / computer assisted manufacturing (CAD/CAM) provide high quality and aesthetic materials. Although CAI and CAD/CAM procedures have facilitated towards a straight forward workflow in the rehabilitation of edentulous patients, immediate implant placement and immediate loading protocols combined are complex and required a high level of organization between the implantologist, the technician and the patient.

The aim of the present case report was to illustrate the feasibility of combined immediate implant placement and loading approach using CAI in the rehabilitation of a patient with a partially dentate mandible asking for a comprehensive treatment and, specifically, not accepting being edentulous all the while.

## Case report

### Initial status und treatment concept

The partially dentate 74-year old patient presented with masticatory problems due a removable partial denture (RPD) with insufficient stability in combination with chronic pain condition in the lower front teeth area. She asked for a comprehensive treatment and did not accept to be edentulous at any stage of the treatment. The patient was a non-smoker and -with the help of antihypertensive (Candecor comp. 32 mg/12,5 mg,) and anticoagulant medication (quick 30; Marcourmar)- in good general health.

**Fig. 1:**
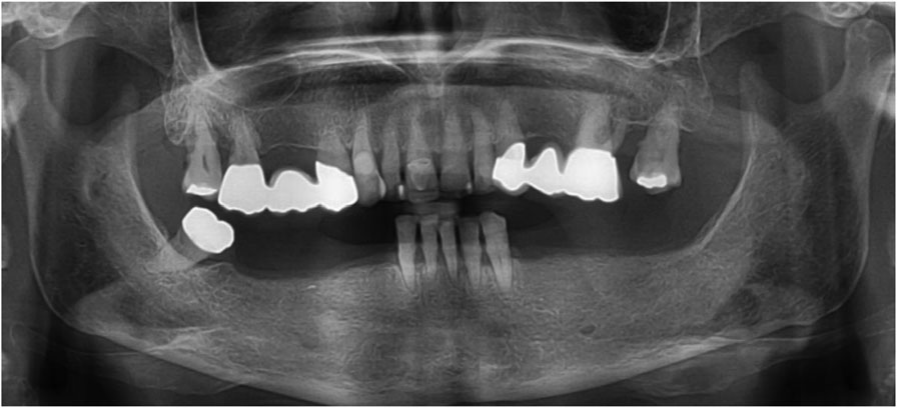
Panoramic radiograph of the initial dental status

**Fig. 2:**
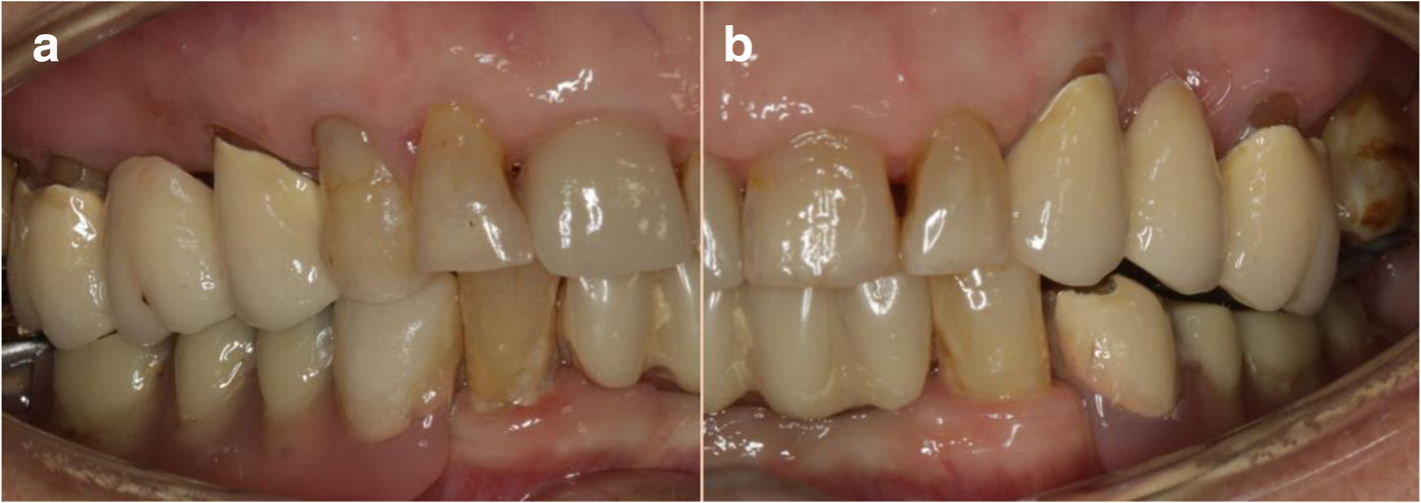
Initial dental status; **a** right side; **b** left side

**Fig. 3:**
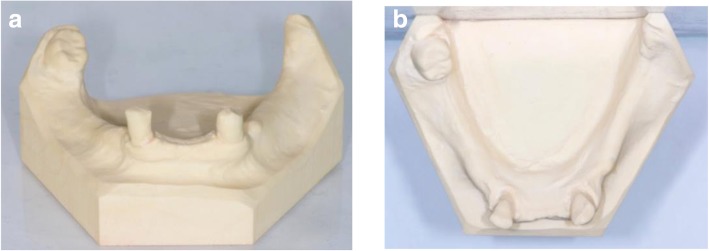
Occlusal and frontal view of the study models after extraction of the 3 incisors 41,31 and 32; **a** front view, **b** occlusal view

The dental status showed an acceptable oral hygiene, some teeth with increased mobility grade III (41/31/32 and 18, 28) and local periodontal problems including horizontal bone loss (42/41/31/32/33, 18/17, 27/28). The teeth 42 and 33 were healthy and not mobile. The alveolar crest in the lateral mandible area showed clinically a wide shape with thick keratinized mucosa. The initial panoramic radiograph revealed stable crestal bone in the lateral mandible area (Figs. 1, 2 and 3). Thus, focusing on the lower jaw, the single tooth prognosis was fair for the teeth 47, 42 and 33 and hopeless for the teeth 41/31/32 [24].

During decision making for the final treatment plan different options were discussed with the patient. Various treatment options including a removable dental prosthesis were discussed with the patient. To keep the patient’s wish for a fixed reconstruction and to never become edentulous in any treatment phase and considering the prognosis of the remaining mandibular teeth, the decision was made to prepare a provisional fixed prosthesis with an immediate loading approach extracting the teeth 42 and 33 for prosthodontic reasons but maintaining tooth 47.

### Digital implant planning (Table 1)

**Fig. 4:**
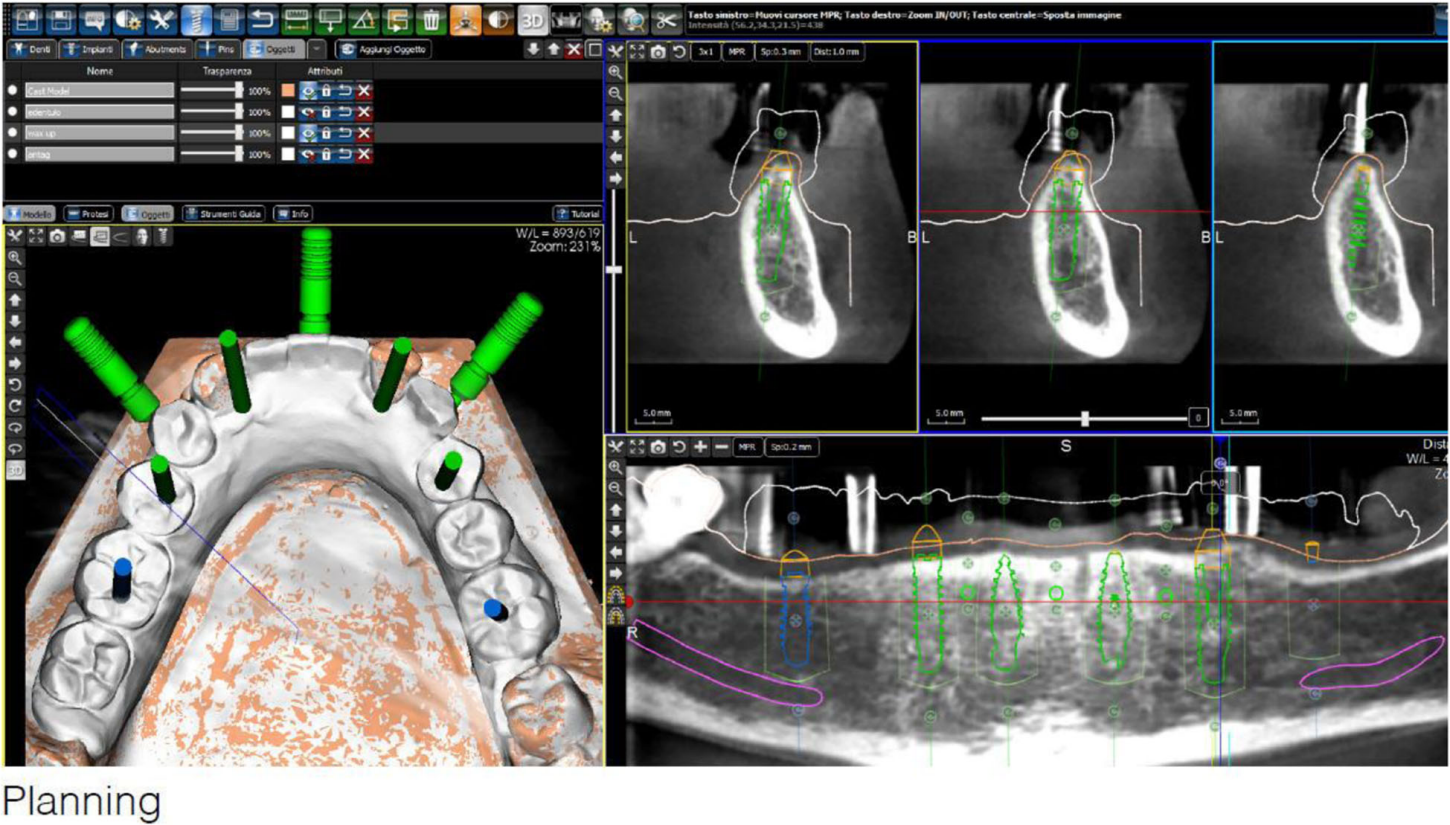
Screen shot of the virtual implant planning (FDA) positions 36,35,33,42,and,46 occlusal, sectional and panoramic views

**Fig. 5:**
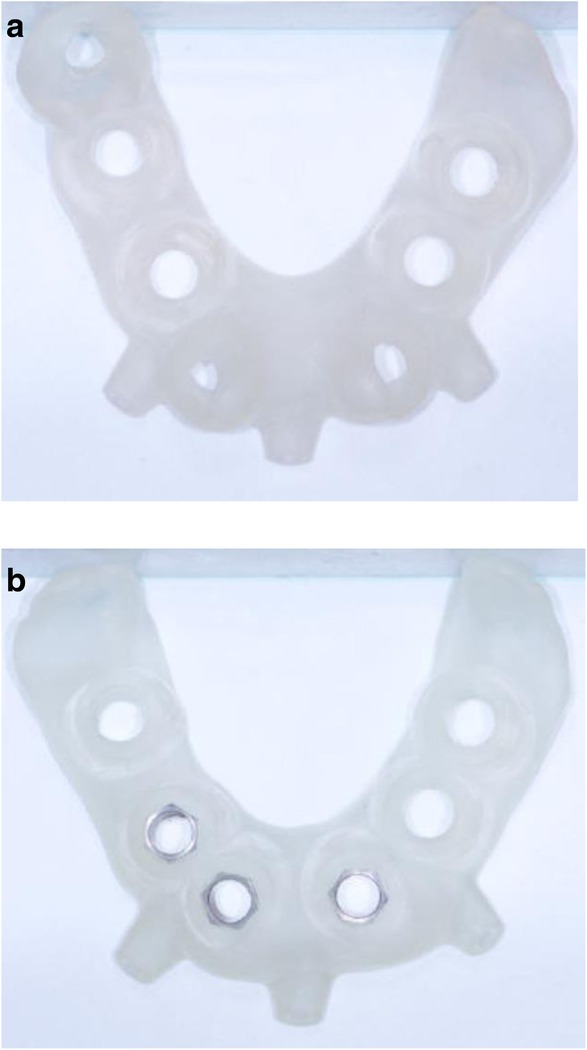
CAD/CAM fabricated surgical guides no. 1 (**a**, tooth and mucosa supported) and no. 2 (**b**, implant and mucosa supported)

**Fig. 6:**
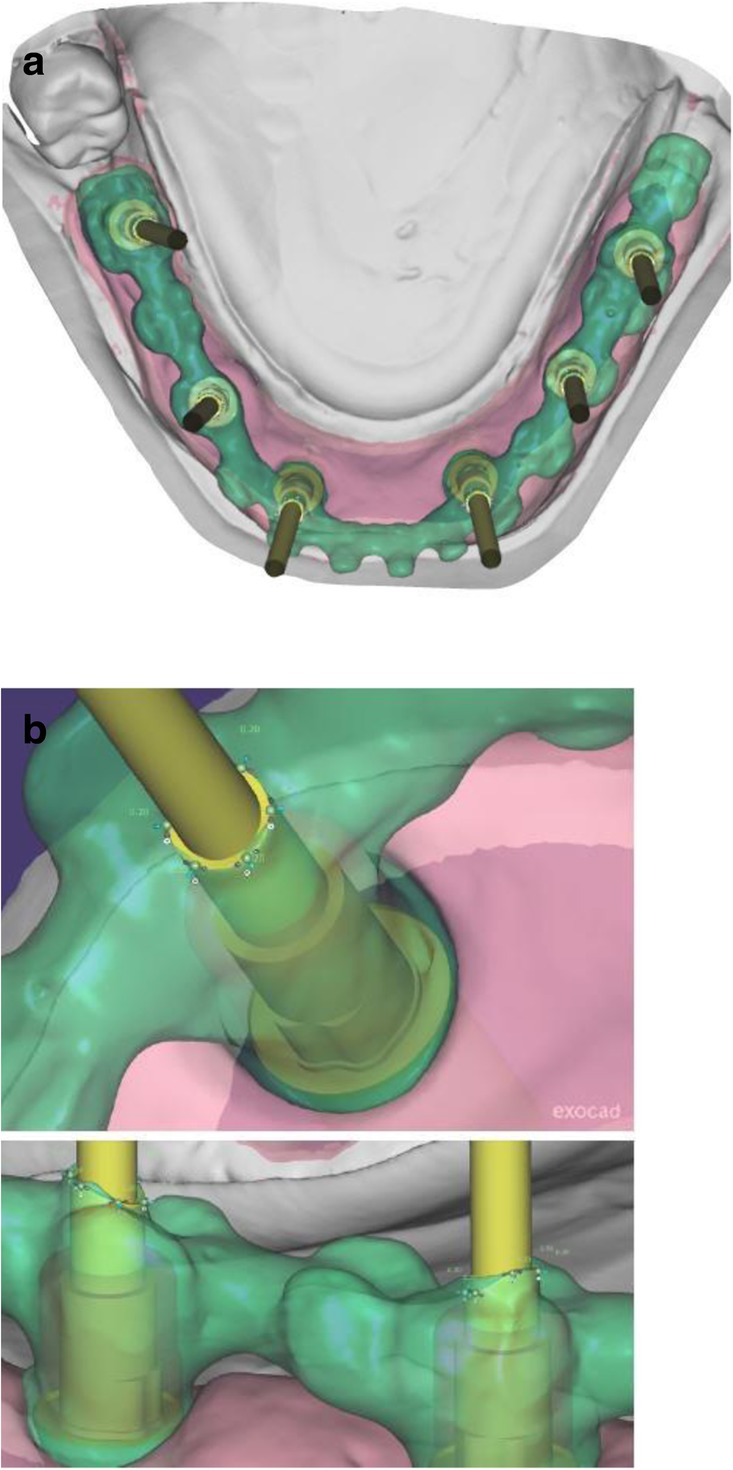
Occlusal view showing CAD of the provisional FDP framework (**a**) and close-up view from the interface between the CAD framework and the prefabricated titanium abutments (**b**)

**Fig. 7:**
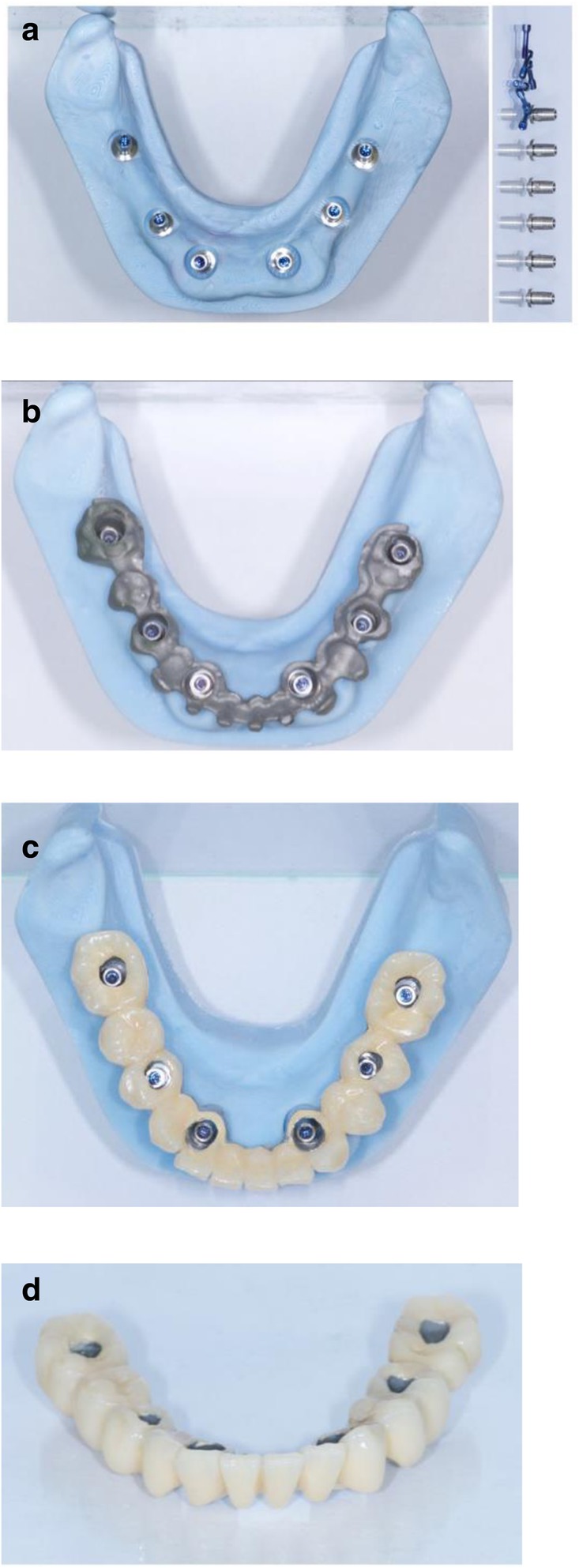
CAD/CAM model with the prefabricated titanium abutments (**a**), the CAD/CAM cobalt-cromium framework (**b**), and the composite veneered provisional FDP (**c**, **d**) before bonding to the abutments

After extraction of the painful and extremely mobile lower front teeth 41/31/32 and adaptation of the existing RPD, a cone beam computed tomography (CBCT) (Pax-Uni 3D, orangedental GmbH & Co. KG, Biberach, Germany) with a 5 × 8 cm field of view and 85KV/5.5 mA/0.2 mm Voxel was performed to proceed with the detailed implant planning (Fig. 4). Based on the anatomical conditions and prosthetic planning (i.e. tooth setup for the provisional RPD), six implants were virtually planned (3Diagnosys, 3DIEMME, Cantu, Italy) in the FDI (Fédération Dentaire Internationale) positions 46, 44, 42, 33, 35, and 36. As the implant positions 42 and 33 interfered with the teeth 43 and 33, a two-step procedure with two surgical templates was planned for the guided implant placement (Fig. 5a, b). The templates were fabricated stereolithographically (DS3000, XFAB, DWS srl, Thiene, Italy) according to the virtual implant planning. Based on the same digital file (Fig. 6a, b) a provisional fixed dental prosthesis (FDP) was prepared preoperatively allowing for an intraoral adaptation between the abutments and the framework to achieve a passive fit (Fig. 7a-d).

**Table 1:** Material and software used for the planning and realization of the treatment

CBCT	Pax-Uni 3D	Orangedental GmbH & Co. KG, Biberach a. d. Riß, Germany
Virtual implant planning	3Diagnosys®	3DIEMME, Cantu, Italy
Implants	Thommen ELEMENT RC 4.5 × 9.5 mm	Thommen Medical AG, Grenchen, Switzerland
CAD	Exocad	Exocad Gmbh, Darmstadt, Deutschland
CAM	M1 Wet	Zirkonzahn, Gais, Italy
Provisional FDP	Prefabricated titanium abutments CAD/CAM CoCr framework Composite veneering & teeth	Thommen Medical AG, Grenchen, Switzerland Sintermetall, Zirkonzahn Srl, Gais, Italy SR Nexco Paste, Ivoclar Vivadent AG, Schaan, Liechtenstein
Final FDP	CAD/CAM CoCr framework Composite veneering & teeth	Sintermetall, Zirkonzahn Srl, Gais, Italy SR Nexco Paste, Ivoclar Vivadent AG, Schaan, Liechtenstein

### Immediate implant placement

During the day of surgery, a single dose of antibiotic (2 g of amoxicillin and clavulanic acid) was administered prophylactically 1 h prior to surgery. This treatment continued for five days (1 g amoxicillin and clavulanic acid twice a day). Prior to the start of surgery, the patient rinsed with 0.2% chlorhexidine for 1 min. Local anesthesia was induced by using a 4% articaine solution with epinephrine 1:100.000.

**Fig. 8:**
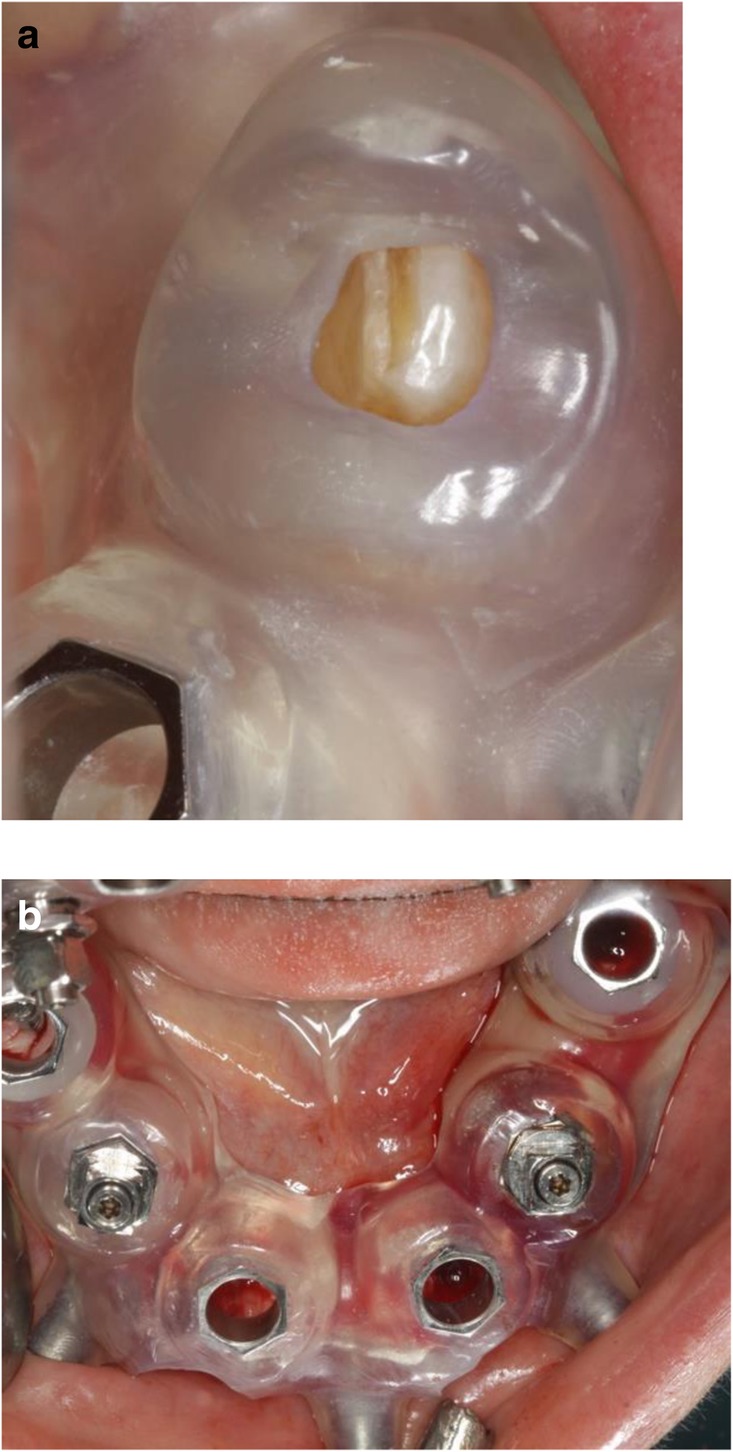
Close-up view of the CAD/CAM guide no. 1 in-situ (tooth and mucosa supported) showing the perfect fit on tooth 33 (**a**), and occlusal view (**b**) of the CAD/CAM guide no. 2 (implant and mucosa supported) after extraction of the teeth 42/33 and placement of the implants 44/35

The two-step approach comprised the flapless guided insertion of the four posterior implants (Thommen Element RC 4.5 × 9.5 mm, Thommen Medical AG, Grenchen, Switzerland), with the first surgical template that was tooth supported (Fig. 8a). The template was then removed and the teeth 42 and 33 previously supporting the guide were extracted. Thereafter, the second surgical template was positioned and stabilized on the four posterior implants with the help of specific abutments and the same anchor pins (Fig. 8b), thus allowing to place the anterior implant 42 and 33 (Thommen Element RC 4.5 × 9.5 mm) guided and immediately after extractions. All the implants were inserted with a torque of 35Ncm and proved good primary stability.

### Immediate loading

**Fig. 9:**
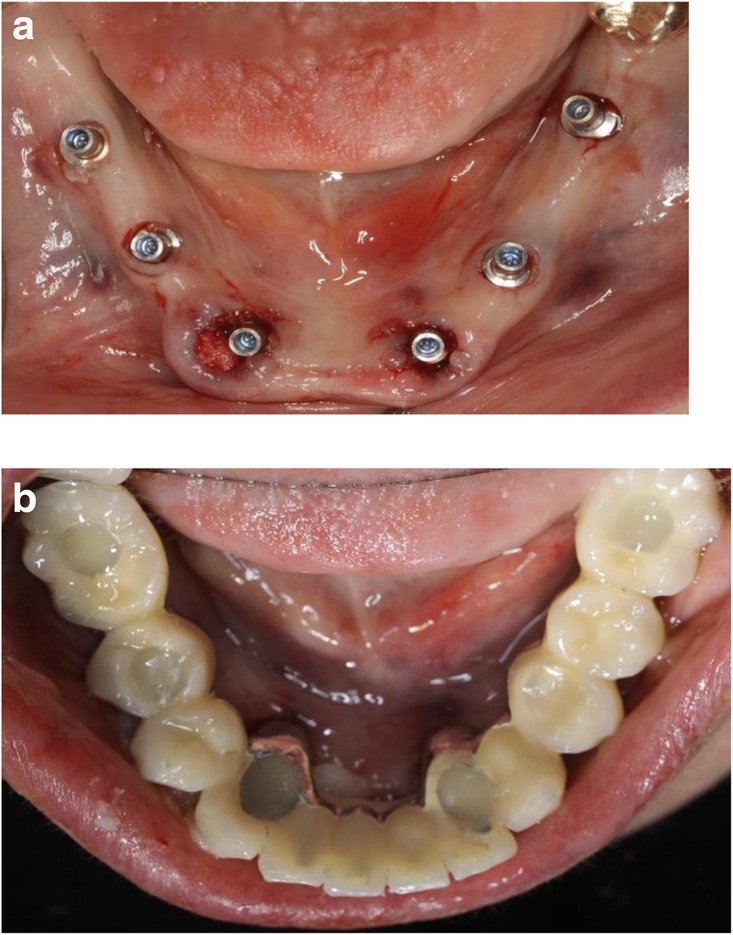
**a**; Occlusal views of the abutments and **b**; the immediate provisional reconstruction that were passively bond in-situ

**Fig. 10:**
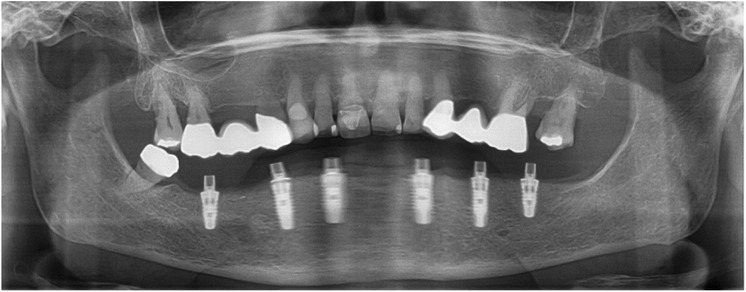
Postoperative panoramic radiograph

After removal of the second surgical template, the standard titanium abutments were mounted on the implants with a torque of 15Ncm (Fig. 9a). The gaps between the abutments and the FDP were filled with Dual-Composite material and the screw retained immediate provisional FDP delivered. The occlusion required only minor adaptations due to the accurate digital preoperative planning (Fig. 9b). The postoperative panoramic radiograph (OPT) showed the parallel axes of the six implants (Fig. 10).

### Final fixed prosthesis

**Fig. 11:**
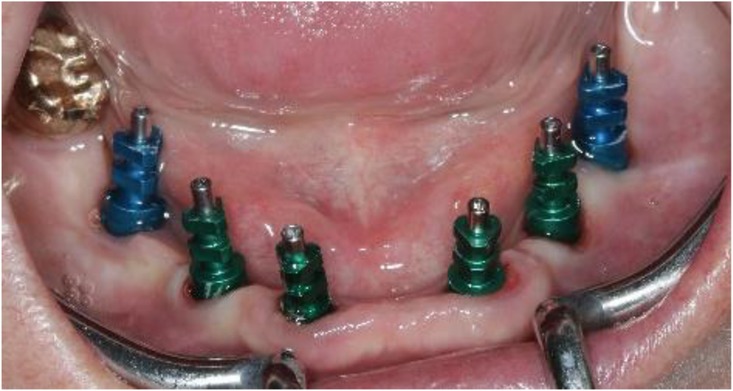
Frontal view showing the screw retained post at impression taking 24 weeks after implant placement

**Fig. 12:**
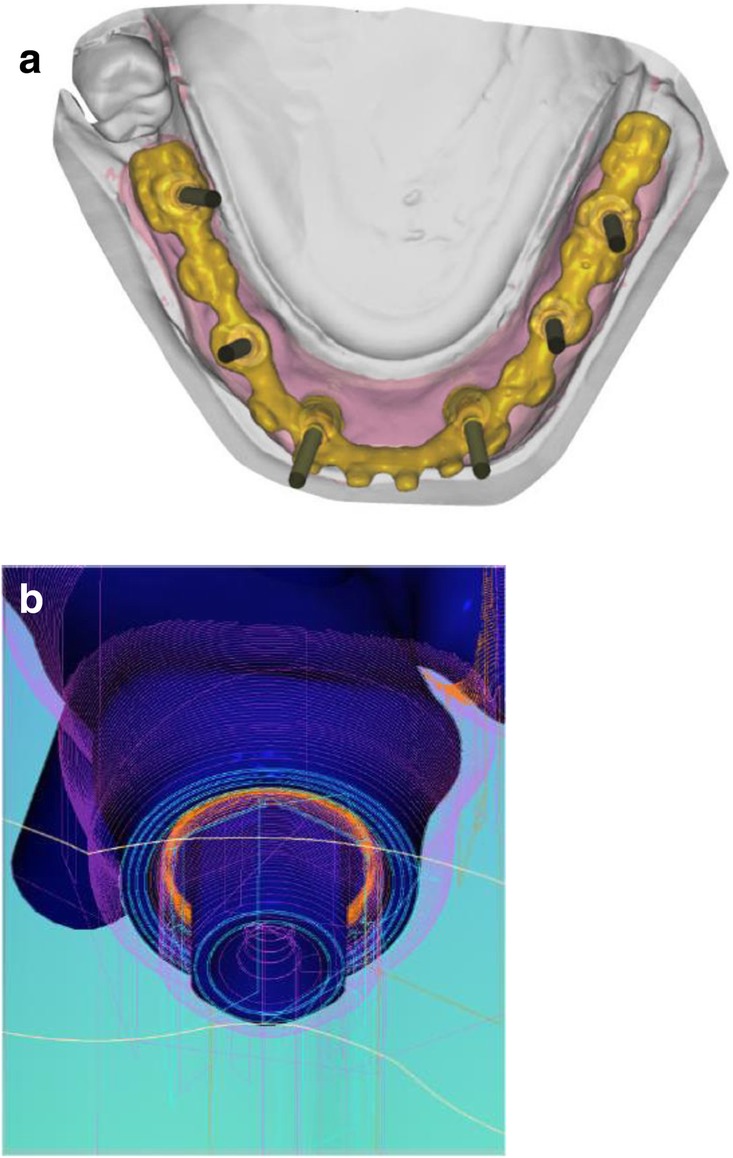
Occlusal view showing CAD of the final FDP (**a**) and detailed screen-shot of the interface geometry (**b**)

**Fig. 13:**
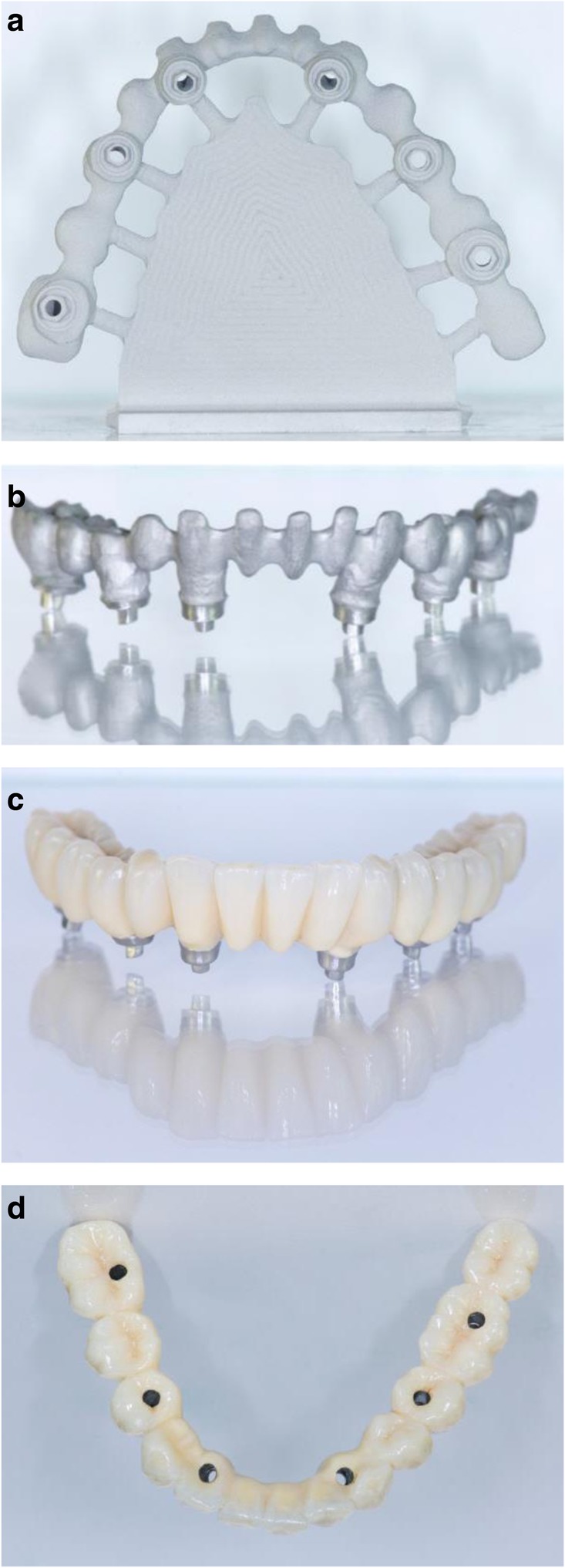
CAD/CAM fabricated one-piece cobalt-cromium framework before (**a**, **b**) and after veneering with composite (Nexco Ivoclar) i.e. final FDP (**c**, **d**)

**Fig. 14:**
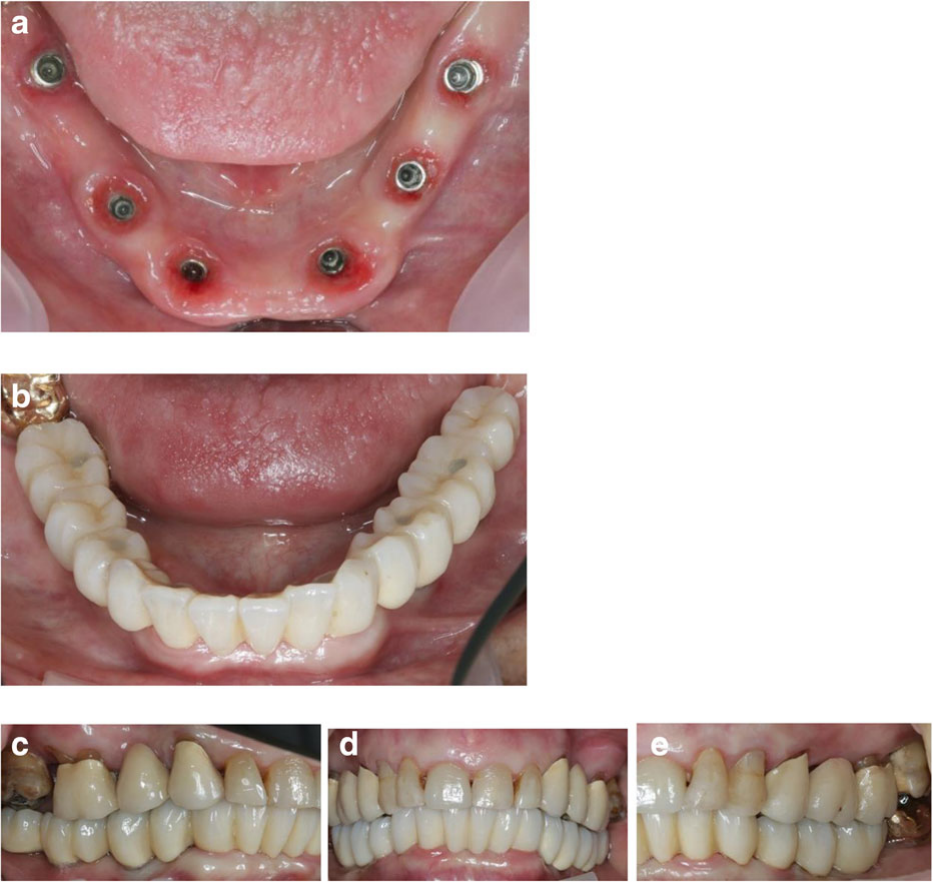
Occlusal, frontal and lateral views at the day of delivery showing healthy peri-implant mucosal conditions (**a**) and the final CAD/CAM reconstruction in situ (**b**-**e**)

**Fig. 15:**
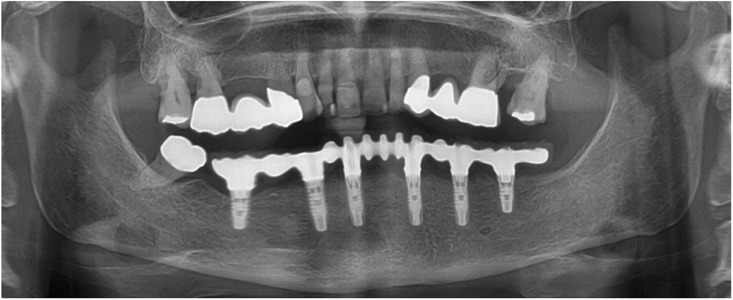
Panoramic radiograph at delivery of the final CAD/CAM FDP

All the six implants osseointegrated successfully without complications. After 6 months with the provisional FDP a conventional impression was taken (screw retained impression copings, open tray technique, polyether material) to fabricate the final FDP on a new precise cast (Fig. 11), which was then digitized with a laboratory scanner (Deluxe Scanner, Open Technologies, Rezzato, Italy). The final framework was designed with straight connection to the implant platforms and with a cut-back allowing for the veneering material (Fig. 12a, b). While the cobalt-chromium framework was fabricated using CAD/CAM technology (Exocad, Exocad gmbH, Deutschland / M1 Wet, Zirkonzahn, Italy) the veneering was performed manually allowing for an individual characterization of the teeth (Fig. 13a-d). The models were fabricated with a laser stereolithography printer (XFAB, DWS srl, Thiene, Italy) using an ABS-like polymer (RD096B, DWS srl, Thiene, Italy). Healthy mucosal conditions were present at the delivery of the final CAD/CAM reconstruction made from cobalt-chromium and composite veneering material (Fig. 14a-f). The accurately fitting FDP was screw retained with 25Ncm and the screw access area covered with composite material. The OPT at the day of delivery showed optimal prosthetic and osseous conditions (Fig. 15). The patient followed a regular maintenance program at the dental hygienist twice a year.

**Fig. 16:**
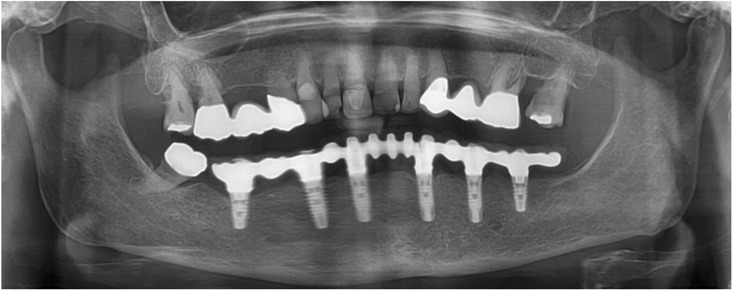
Panoramic radiograph at the 12-months recall appointment

At the one year follow-up appointment, healthy mucosal and stable crestal peri-implant conditions could be observed (Fig. 16). The patient was very pleased with the esthetic and functional outcome. Thus, the performed treatment was successful and showed stable results without complications or need for maintenance service after the first year.

## Discussion

The use of CAI software in the preoperative virtual three-dimensional implant planning allowed for guided and immediate implant placement, and proved to be especially beneficial in the presented mandibular full-arch case. While there are some studies that investigated outcomes of immediately loaded implants placed in edentulous patients using computer-assisted template-guided surgery to support a FDP [25], only few case reports are available in literature describing the entire workflow, the patients state in detail, and the usage of guided surgery templates with subsequent immediate loading [3, 4]. The considerably more complex combination of immediate implant placement and immediate loading required a high level of organization between implantologist and technician, minimizing patient’s compliance. Pozzi et al. reported excellent results with CAD/CAM cross-arch Zirconia bridges on immediately loaded implants placed with computer-assisted/template-guided surgery [26]. Several investigators presented analyses of recent studies in this context elaborating the factors that influence mainly the accurate implant placement but also the comparable outcome of the reconstructions after guided implant placement [15, 20, 21, 27–31]. In the present case report, two CAD/CAM surgical templates were combined in this partially dentate patient with extraction of the teeth 42/33 and immediate implants performed in a sequenced order. The first scanner-based template was teeth and mucosa supported enabling a higher template stability and, thus, more accurate guided osteotomies and implant placement. Four posterior implants were placed with this approach allowing to support the second surgical template after extraction of the anterior teeth 42/33. The stability on these four points was high as the implants 42/33 showed a torque value of 35 to 40 Ncm each. The subsequent two anterior immediate implants were thus placed perfectly guided. Different factors contributed to this insertion torque such as the depth of the planned implant position in a more apical area than the extraction site, the minimally invasive tooth extraction, the macroscopic implant geometry and the osteotomy protocol with a smaller drilling diameter compared to the implant diameter (as proposed by the company), the accurate performance of the single steps in the pre- and intraoperative phases, and the bone density in the anterior mandibular area. The prefabricated provisional FDP was prepared to connect the abutments to the FDP intraorally, which was easily to be performed given the accurate result of the implant positions. With this approach the passive fit of the FDP was maximized, the clinical chairside efforts (in terms of abutment connection and occlusal adaptations) were minimal and the predictability was very high compared to different limitations and problems reported in a recent review [32].

The preoperative communication between the dentist and the technician during the decision making and planning phase were essential for the concise timing in the clinic, ensuring highest surgical and prosthodontic performance accuracy in this particular case. Therefore, up-to-date software and hardware with the knowledge to apply information to the specific products was required. This case report supports the need for minimally traumatic or flapless surgery, optimal implant positioning and immediate loading, as summarized in a recent review on randomized controlled trials [33].

## Conclusions

The present case report emphasized the efficient workflow and the predictable outcome using computer assisted implantology. The fabrication of an immediate provisional FDP and, subsequently, the final CAD/CAM reconstruction was facilitated by CAI fulfilling patient’s wish of being continuously restored during the complete treatment.

## Abbreviations

CAD/CAM: Computer assisted design / computer assisted manufacturing
CAI: Computer assisted implantology
CBCT: Cone beam computed tomography
CT: Computerized tomography
FDI: Fédération Dentaire Internationale
FDP: Fixed dental prosthesis
RPD: Removable partial denture
